# Supplementary material for: Splice-Junction-Based Mapping of Alternative Isoforms in the Human Proteome
Source: Cell Rep. Author manuscript; Available in PMC 2020 Jan 15. (PMC6961840; doi:10.1016/j.celrep.2019.11.026)
Supplement: 2 [file NIHMS1546469-supplement-2.zip › DF1/PXD010154/Heart/Heart_49_MAP4_MYHDDDLADLVFPSSATADTSIFAGQNDPLKDSYVPLELAK.pdf]

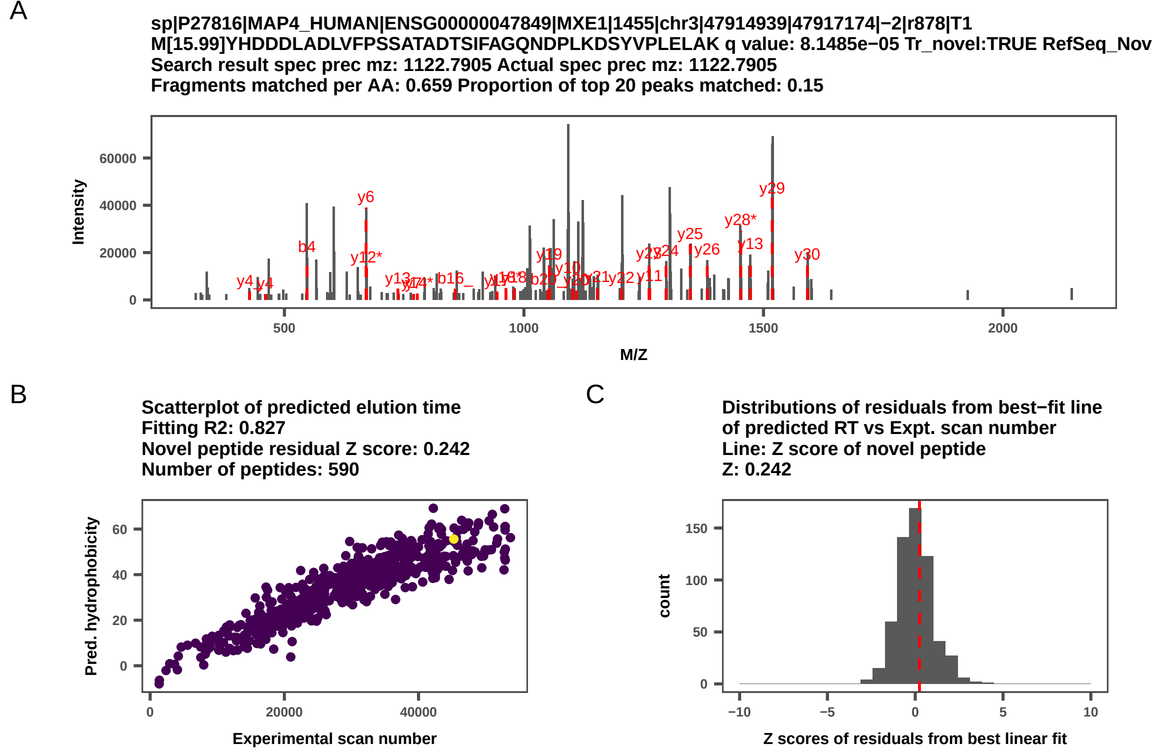
A
sp|P27816|MAP4_HUMAN|ENSG00000047849|MXE1|1455|chr3|47914939|47917174|−2|r878|T1
M[15.99]YHDDDLADLVFPSSATADTSIFAGQNDPLKDSYVPLELAK q value: 8.1485e−05 Tr_novel:TRUE RefSeq_Nov
Search result spec prec mz: 1122.7905 Actual spec prec mz: 1122.7905
Fragments matched per AA: 0.659 Proportion of top 20 peaks matched: 0.15
Intensity
60000
40000
20000
0
y6
b4
y12*
y29
y28*
y25
y26
y13
y30
y19
y10
M/Z
500
1000
1500
2000
B
Scatterplot of predicted elution time
Fitting R2: 0.827
Novel peptide residual Z score: 0.242
Number of peptides: 590
Pred. hydrophobicity
60
40
20
0
0
20000
40000
Experimental scan number
C
Distributions of residuals from best−fit line
of predicted RT vs Expt. scan number
Line: Z score of novel peptide
Z: 0.242
count
150
100
50
0
−10
−5
0
5
10
Z scores of residuals from best linear fit
